# *Chlamydia trachomatis *growth inhibition and restoration of LDL-receptor level in HepG2 cells treated with mevastatin

**DOI:** 10.1186/1476-5926-9-3

**Published:** 2010-01-28

**Authors:** Yuriy K Bashmakov, Nailya A Zigangirova, Yulia P Pashko, Lidia N Kapotina, Ivan M Petyaev

**Affiliations:** 1 Cambridge Theranostics Ltd, Babraham Research Campus, Babraham, Cambridge, CB2 4AT, UK; 2 Department of Medical Microbiology, Institute of Epidemiology and Microbiology RAMS, 18 Gamaleya Str, Moscow 123098, Russia

## Abstract

**Background:**

Perihepatitis is rare but consistently occurring extragenital manifestation of untreated *Chlamydia trachomatis *infection. Despite of possible liver involvement in generalized C. *trachomatis *infection, the ability of the pathogen to propagate in the hepatic cells and its impact on liver functions is not thoroughly investigated. The effect of mevastatin, an inhibitor of 3-hydroxy-3-methylglutaryl CoA reductase, on *C. trachomatis *growth in human hepatoma cell line HepG2 has been studied. Bacterial growth was assessed by immunostaining with FITC-labeled monoclonal antibody against chlamydial lipopolysaccharide and by RT-PCR for two chlamydial genetic markers (16S rRNA and euo).

**Results:**

Chlamydial inclusion bodies were seen in approximately 50% of hepatocytes at 48 hours in the post infection period. Lysates obtained from infected hepatocytes were positive in the infective progeny test at 48 and especially in 72 hours after infection initiation. It has been shown that chlamydial infection in hepatocytes also leads to the decline of LDL-receptor mRNA which reflects infection multiplicity rate. Additions of mevastatin (1, 20 and 40 μM) 1 hour before inoculation restored and upregulated LDL-receptor mRNA level in a dose-dependent manner. Mevastatin treatment had no effect on internalization of chlamydial particles. However it reduced drastically the number of chlamydial 16S rRNA and euo transcripts as well as overall infection rate in HepG-2 cells. Complete eradication of infection has been seen by immunofluorescent staining at 40 μM mevastatin concentration, when expression level of chlamydial 16S rRNA and euo was undetectable. Lower concentration of mevastatin (20 μM) promoted euo expression level and the appearance of atypically small chlamydial inclusions, while there was a noticeable reduction in the number of infected cells and 16S rRNA transcripts.

**Conclusions:**

*C. trachomatis *can efficiently propagate in hepatocytes affecting transcription rate of some liver-specific genes. Ongoing cholesterol synthesis is essential for chlamydial growth in hepatocytes. Inhibitors of cholesterol biosynthesis can supplement conventional strategy in the management of *C. trachomatis *infection.

## Background

*Chlamydia trachomatis *is a prevalent bacterial pathogen causing most of the cases of urogenital infections and preventable blindness in the world. Epididymitis and urethritis in men, cervical as well as the urethral inflammation in woman may lead to acute pelvic inflammatory disease and variety of other extragenital manifestations in both sexes. Among most frequent extragenital manifestations of *C. trachomatis *are sexually acquired reactive arthritis (SARA), conjunctivitis and perihepatitis [1]. In most of the cases of ophthalmological manifestations *C. trachomatis *can be detected and/or isolated in the eye swabs [2]. It is believed that immunological and hormonal phenotype as well as some genotype characteristics, particularly expression of human leucocyte antigen B27, predetermine the severity of extragenital manifestations caused by *C. trachomatis *[3]. Delayed cell-mediated immunological response is also known to play an important role in the systemic generalization of chlamydial disease [4].

However there is a growing body of evidence that *C. trachomatis *can be present and isolated from extragenital tissues and organs. Bacterial antigens, DNA and/or RNA can be detected in whole blood [5,6] since *C. trachomatis *can efficiently propagate in mononuclear cells [7] as well as in astrocytes [8], muscle cells [9] and myocardiocytes [10]. Virulent forms of *C. trachomatis *can be isolated from synovial exudate [11], ascitic fluid [12,13], liver biopsy material [14], and respiratory secretion fluids [15]. Similar pattern of extragenital manifestations has been reported in animal experiments.

Lesions containing virulent *C. trachomatis *have been reported in lungs, liver and spleen of BALB/c mice in the post-infection period [16]. With the exception of a single report [14] there are no confirmed cases of *C. trachomatis *isolation from the human liver or any well articulated insights on the potential role of chlamydial infection in hepatobilliary pathology. However, recently shown ability of *C. trachomatis *to propagate in hepatocytes [17,18] leads to many questions about possible involvement of liver in systemic chlamydial disease.

In the present paper we have investigated the infectability of *C. trachomatis *toward immortalized human hepatoma cells (HepG2 cell line) and some metabolic consequences of chlamydia propagation in the hepatic cell line. In particular, of mRNA regulation of major lipogenic genes in the host cells and effect of mevastatin, an inhibitor of 3-hydroxy-3-methyglutaryl CoA reductase (HMG-CoA reductase), in cases of chlamydial infection in HepG2 cells are reported below.

## Methods

### Reagents

All reagents were purchased from Sigma-Aldrich unless specifically mentioned otherwise. HepG2 and Hep2 cells were obtained from "European Collection of Cell Cultures" (Salisbury, UK).

### Cell culture and organisms

HepG2 cells were cultured in 5% CO_2 _in DMEM supplemented with 10% Fetal Bovine Serum (FBS) and 2 mM glutamine. Cells were grown in 6, 24, and 96 well plates until confluence rate of 80% was reached. Addition of mevastatin at concentrations ranging from 1 μM to 40 μM was done 1 hour before inoculation of *C. trachomatis*. Strain L2/Bu434 of *C. trachomatis *was kindly provided by Dr. P. Saikku (University of Oulu, Finland). Chlamydial strains were initially propagated in Hep2 cells and purified by Renografin gradient centrifugation as described [19]. Chlamydial titers were determined by infecting Hep2 cells with 10-fold dilutions of thawed stock suspension. Purified elementary bodies (EB) with known titer were suspended in sucrose-phosphate-glutamic acid buffer [19] and used as inoculums for HepG2 cells.

HepG2 plates were infected with *C. trachomatis *at multiplicities of infection (MOI) of 1 or 2 in DMEM with 0.4% glucose without FBS and cycloheximide and centrifuged for 0.5 hour at 1500 g. The cells were harvested for RNA analysis in 24 hours (expression of chlamydial genes) and in 48 hours (expression of eukaryotic genes and immunofluorescence analysis) after infection after the inoculation of *C. trachomatis*. Acell viability assay was conducted routinely for each group of the experiment using 2% trypan blue exclusion test. The cell monolayers with viability > 85% were used for RNA extraction and/or immunostaining. There was a significant decrease in number of viable hepatocytes during the late stage of chlamydial infection in HepG2 cells (72 hours).

### Immunofluoresence staining

Infected HepG2 monolayers grown 48 hours on coverslips in 24 well plates, which were fixed with methanol. Permeabilized cells were stained by direct immunofluorescence using FITC - conjugated monoclonal antibody against chlamydial lipopolysaccharide (NearMedic Plus, RF). Inclusion-containing cells were visualized using Nikon Eclipse 50 i microscope fluorescence microscope at X1350 magnification.

### Internalization assay

Internalization assay has been performed as described [20]. Briefly, to visualize attachment of *C. trachomatis *to HepG2 cells, elementary bodies (EB) of *C. trachomatis *were added at MOI 50 to the 24 well plates with coverslips containing hepatocytes monolayers. The EB were allowed to attach in presence or absence of 40 μM mevastatin for 60 min at 4°C after which the inoculum was removed, cell were washed 3 times with ice-cold PBS. To visualize attached particles, the cell monolayers were fixed in 4% paraformaldehyde for 15 min on ice. This regimen of fixation is believed to maintain the integrity of the plasma membrane in the host cells [20]. After fixation the cells were washed with PBS and incubated for 30 min with monoclonal chlamydial LPS-specific antibody labeled with FITC (1 μg/ml, NearMedic Plus, RF) for visualization of attached particles. Internalization has been studied in separate set of experiments. To allow attachment, HepG2 cells were incubated with EB of *C. trachomatis *in presence or absence of 40 μM mevastatin for 1 hour at 4°C after which the inoculum was removed and the cells were washed 3 times with ice-cold PBS. The cells were transferred to 37°C for 1 hour to permit internalization. After fixation with 4% paraformaldehyde (15 min, room temperature) the cells were incubated for 30 min with the polyclonal antibody raised against EB of *C. trachomatis *(Gamaleya Institute of Microbiology and Epidemiology, Moscow, RF). This step was performed in order to block attachment sites of non-internalized EB. After fixation with methanol (15 min, room temperature), which allows penetration of antibody inside of the cells [20], cell monolayers were incubated for 30 min with 1 μg/ml of monoclonal FITC-conjugated antibody against *C. trachomatis *major outer membrane protein (MOMP) (NearMedic Plus, RF). The cells were washed thoroughly with PBS and analyzed by immunofluorescent microscope.

### Assessment of infective progeny

In order to assess the infective progeny accumulation in HepG2 cells after 48 hour cultivation period, HepG2 cells were harvested, frozen and thawed, as described elsewhere. Serial dilutions of lysates were inoculated onto Hep-2 cells and centrifuged for 0.5 hour at 1500 g. The infected cells were visualized with *C. trachomatis *LPS-specific antibody in 48 hours of the post-infection period.

### RNA extraction and reverse transcription

RNA was isolated from HepG2 monolayers grown on 6-well plates using TRIZol (Invitrogen). Total mRNA pretreated with DNase I (DNA-free™, Ambion) and quantified on the spectrophotometer NanoDrop ND-100 (ThermoFisher Scientific, Wilmington, USA) was converted into cDNA using random hexamer primers and a SuperScript III First-Strand Synthesis Kit (Invitrogen, Karlsruhe, Germany).

### Quantitative real-time PCR

The mRNA levels for two different developmental genes of *C. trachomatis *were analyzed in HepG2 cells by quantitative RT-PCR using thermocycler ANK 32 (Syntol, RF). The 16S rRNA and gene encoding DNA-binding protein Euo were studied as constitutive markers of the early stage of chlamydial developmental cycle. Primers for *C. trachomatis *16S rRNA (sense - 5'-GGCGTATTTGGGCATCCGAGTAACG-3', antisense - 5'-TCAAATCCAGCGGGTATTAACCGCCT-3') and *C. trachomatis *Euo (sense - 5'-TCCCCGACGCTCTCCTTTCA-3', antisense - 5'-CTCGTCAGGCTATCTATGTTGCT-3') were verified and used under thermal cycling conditions - 95°C for 10 min and 50 cycles of 95°C for 15 seconds, 60°C for 1 min and 72°C for 20 seconds. Serial dilutions of *C. trachomatis *RNA, extracted from chlamydia-infected Hep-2 cells, were used as a standard for quantification of chlamydial gene expression. The results of PCR analysis for chlamydia-specific genes were normalized to mRNA values of human beta actin (β-actin, primers: sense - 5'-GCACCCAGCACAATGAAGAT-3', antisense - 5'-GCCGATCCACACGGAGTAC-3'). Among other human-specific genes studied were major lipogenic enzymes: 3-hydroxy-3-methyglutaryl CoA reductase (HMG CoA reductase, primers: sense-5'-CAAGGAGCATGCAAAGATAATCC-3' antisense -5'-GCCATTACGGTCC CACACA-3'); 3-hydroxy-3-methyglutaryl CoA synthase (HMG CoA Syn, primers: sense - 5'-GACTTGTGCATTCAAACATAGCAA-3', antisense - 5'-GCTGTAGCAGGGAGTCTTGGTACT-3'); squalene synthase (SS, primers: sense - 5'-ATGACCATCAGTGTGGAAAAGAAG-3', antisense - 5'-CCGCCAGTCTGGTTGGTAA-3'); and fatty acid synthase (FAS, primers: sense-5'-TCGTGGGCTACAGCATGGT-3', antisense - 5'-GCCCTCTGAAGTCGAAGAAGAA-3').

The mRNA levels for lipogenic enzymes as well as mRNAs for LDL-receptor (LDL-R, primers: sense - 5'-GGCTGCGTTAATGTGACACTCT-3', antisense - 5'-CTCTAGCCATGTT GCAGACTTTGT-3') and LDL-receptor related protein (LRP, primers: - 5'-CCTACTGGACGCTGA CTTTGC-3' antisense - 5'-GGCCCCCCATGTAGAGTGT-3') in the host cells were normalized to human β-actin expression level. The mRNA expression levels in the host cells were referenced to the CT values in uninfected HepG2 cells grown at the same conditions. That reference value was taken as 1.00. Each cDNA sample was tested by PCR at least three times. All experiments were repeated at least twice. Representative sets of results are shown below.

## Results

### *C. trachomatis *growth in HepG2 cells

Immunofluorescent images of HepG2 infected cells reveal that C. trachomatis can efficiently grow in immortalized hepatocytes cells line. Positive immunofluorescence was first apparent within 24 hours of post-infection period and did not differ in intensity at MOIs of 1 and 2. Inclusion bodies were seen in about 50% of cells at 48 hours in the post-infection period at MOI of 1. Up to 70% of the infected cells were seen at multiplicity rate of 2. Most of the immunostaining was localized throughout whole cytoplasm. However some cells had perinuclear pattern of immunofluorescence with no intranuclear inclusions seen. At 48 and especially 72 hours of the post-infection period, immunostaining was stronger with numerous inclusion bodies. Some of them were released from the ruptured cells. To determine if C. trachomatis can be cultured from HepG2 monolayers, we harvested 24 and 48 hour cultures of hepatocytes. Replication was not observed when 24 hour lysates of hepatocytes were inoculated to Hep2 cells. However the lysates obtained in 48 and especially 72 hour were positive in the infective progeny test.

### LDL-receptor mRNA and multiplicity of infection

As can be seen from Table 1, 48 hour propagation of *C. trachomatis *in HepG2 cells did not affect mRNA for a major housekeeping gene - 36B4, nor mRNAs for lipogenic enzymes. However, there is dose-dependent decline in LDL-receptor mRNA, reflecting multiplicity infection level. LDL-receptor related protein mRNA remained unchanged.

**Table 1:** Folds and mRNA changes in HepG2 cells infected with *C. trachomatis *at different infectivity rates.

Parameter	Non-infected cells	Infected cells	
		MOI 1	MOI2
36B4^ct^	18.37	18.26	18.01
HMG-CoA Red	1	1.31	0.98
HMG-CoA Synth	1	1.06	0.87
SS	1	1.21	0.89
LDL-R	1	0.76	0.56
LRP	1	0.87	0.99
FAS	1	0.88	0.89

HepG2 cells were set up, grown and infected with *C. trachomatis *in presence or absence of mevastatin as described in Methods. RNA was extracted in 48 hours after inoculation of the bacteria. RNA levels for the genes of interest were normalized to 36B4 expression level, whose CT values are represented in the upper row of the Table. All RNA values in the infected cells are referenced to non-infected control

### Mevastatin reverses LDL-receptor mRNA decline

Inhibitors of HMG-CoA reductase are the most powerful activators of LDL receptor, whose activity on the LDL-receptor is mediated by SREBP pathway [21]. The addition of mevastatin to HepG2 cells infected with *C. trachomatis *at MOI of 1 did not affect cell viability nor mRNA levels of 36B4 (Table 2). However, LDL-receptor mRNA level was dose-dependently upregulated with the increasing concentrations of mevastatin, reaching 2 fold induction at 40 μM level. This effect was even more pronounced at 72 hours of the post-infection period though cell viability was declining (results not shown). There is also dose-dependent upregulation of cholesterologenic enzymes (HMG-CoA reductase, HMG-CoA synthase, SS) which is well known effect of statins in the cultures cells [22]. Notably, LDL-receptor related protein mRNA was not impacted under all conditions studied.

**Table 2:** Folds and mRNA changes in *C. trachomatis*-infected HepG2 cells after addition of mevastatin.

		Infected cells -- Addition of mevastatin			
Parameter	Non-infected cells	0 μM	1 μM	20 μM	40 μM
36B4^ct^	16.94	17.04	16.94	16.98	17.01
HMG-CoA Red	1	1.06	1.17	1.7	1.81
HMG-CoA Synth	1	0.79	1.46	1.53	1.89
SS	1	0.87	1.27	1.54	1.73
LDL-R	1	0.69	1.38	1.63	2.08
LRP	1	1.09	0.85	0.91	0.99
FAS	1	0.95	0.92	0.89	0.96

HepG2 cells were set up, grown and infected with *C. trachomatis *in presence or absence of mevastatin as described in Methods. RNA was extracted in 48 hours after inoculation of the bacteria. RNA levels for the genes of interest were normalized to 36B4 expression level, whose CT values are represented in the upper row of the Table. All RNA values in the infected cells are referenced to non-infected control.

### Mevastatin inhibits chlamydial growth in HepG2 cells

**Figure 1:**
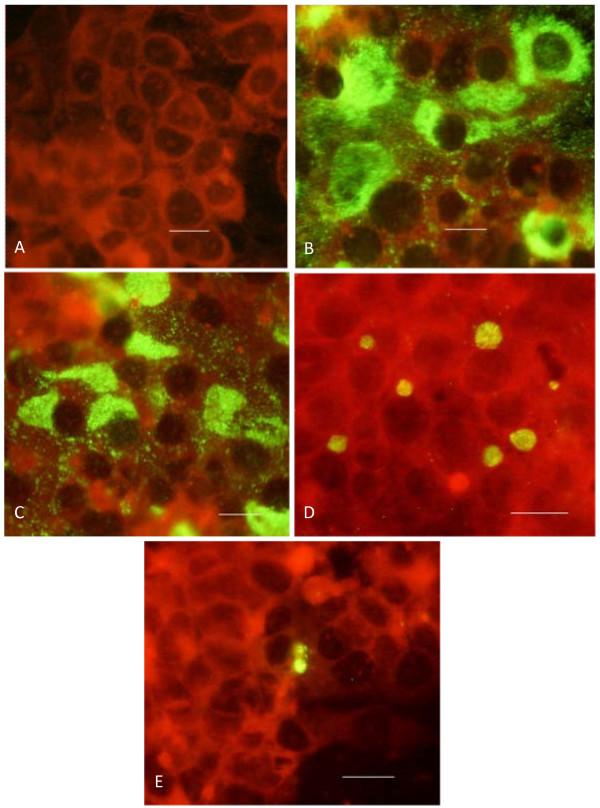
**Immunofluorescent images of HepG2 cells infected with *C. trachomatis *in presence of mevastatin**. HepG2 cells were set up, grown and infected with *C. trachomatis *in presence or absence of mevastatin as described in Methods. Immunofluorescence analysis was performed 48 hours after inoculation of the pathogen. **A **- non-infected cells; **B **-- infected cells with no mevastatin; **C **-- infected cells in presence of 1 μM mevastatin: **D **-- infected cells in presence of 20 μM mevastatin; **E **-- infected cells in presence of 40 μM mevastatin. Scale bar = 10 μm.

**Figure 2:**
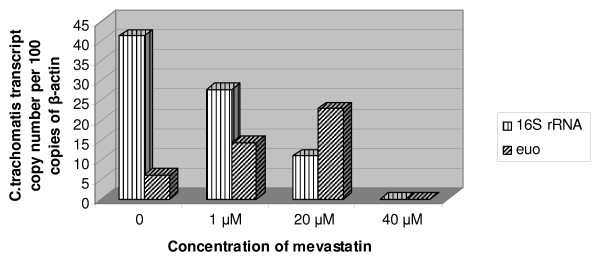
**Expression of chlamydial 16S RNA and euo in infected hepatocytes grown at different concentration of mevastatin**. HepG2 cells were set up, grown and infected with *C. trachomatis *in presence or absence of mevastatin as described in Methods. RNA was extracted in 24 hours after inoculation of the bacteria. Expression of chlamydial genes was normalized to copy number of eukaryotic β-actin.

Figure 1 shows representative immunofluorescent images of HepG2 cells infected with *C. trachomatis *in presence of increasing concentrations of mevastatin. As can be seen, the effect of mevastatin was marginal at the concentration of 1 μM, though some decline in the number of infected cells has been noticed. However, 20 μM mevastatin reduced both the number of inclusion bodies in the infected cells, promoting a perinuclear pattern of staining. Mevastatin-treated cells (20 μM) appeared to contain smaller inclusion bodies similar to those that occur during persistent chlamydial infection [23]. The highest concentration of mevastatin tested (40 μM) abolished the number of infected cells almost completely. Analysis of bacterial transcripts showed a similar tendency. As can be seen from Figure 2, 16S rRNA and euo mRNA were undetectable at highest concentration of mevastatin used, whereas at 20 μM and 1 μM mevastatin reduced the expression level for 16S rRNA by 8 and 3 fold respectively. There is significant induction of euo mRNA at 20 μM mevastatin concentration.

**Figure 3:**
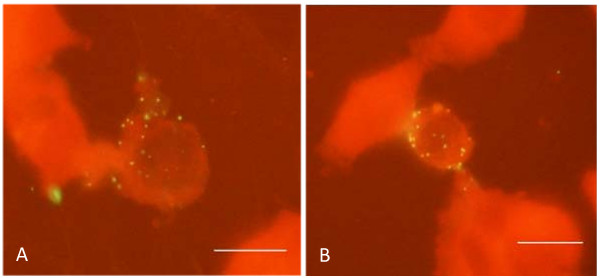
**Attachment of chlamydial particles to plasma membrane of hepatocytes in presence or absence of mevastatin**. HepG2 cells were set up, grown and incubated with chlamydial particles (EB) in presence or absence of mevastatin as described in Methods. Attached particles were visualized with FITC-labeled antibody against chlamydial LPS. **A **-- attachment of chlamydial particles in absence of 40 μM mevastatin: **B **-- attachment of chlamydial particles in presence of 40 μM mevastatin. Scale bar = 10 μm.

Inhibition of chlamydial growth in cultured cells in presence of mevastatin may take place due to abnormal internalization of chlamydial particles, since the entry of chlamydial particles into mammalian cells requires interaction of pathogens with lipid rafts of plasma membrane [24]. Therefore, we next investigated the internalization rate of chlamydial particles into HepG2 cells in presence of 40 μM mevastatin. As can be seen from Figure 3, HepG2 cells treated with 40 μM mevastatin have similar number of chlamydial particles attached to the plasma membrane when compared to untreated control cells. Mevastatin treatment did not affect the number of internalized particles as well (results not shown).

## Discussion

Although there is a small but growing body of evidence that *C. trachomatis *can be disseminated widely throughout the human body, the physiological consequences and overall medical relevance of extragenital propagation of *C. trachomatis *remains poorly understood. First of all, our results confirm initial observations [25] showing the ability of *C. trachomatis *to propagate in HepG2 hepatoma cell line. More importantly, we have demonstrated that propagation of *C. trachomatis *in hepatocytes follows full infectious cycle leading to the formation of infectious progeny in 48 and 72 hours of post-infection period. Propagation of the pathogen distinctively affects some specific functions of the liver cells. In particular, *C.trachomatis *ameliorates transcription of LDL-receptor in hepatocytes, which may have various consequences for lipid homeostasis.

Chlamydial organisms are strict intracellular parasites, whose requirements in the metabolites are covered by the host cells. Enhanced uptake of the substrates and metabolites by the infected host cells is a well known "signature" strategy of chlamydial infection mandatory for successful accomplishment of its infectious cycle [25]. However, in the case of the chlamydial growth in HepG2 cells we have seen significant decline in LDL-receptor mRNA, which may potentially result in the reduction of lipid uptake. The biological significance of this finding remains unclear. However it is possible to assume, that decline in the LDL-receptor mRNA might represent a mechanism of metabolic adaption of the host cell to chlamydial infection targeted on limitation of lipid supply and chlamydial growth in the cells. Unfortunately we were not able to document corresponding changes in LDL-receptor protein level due to decline in number of viable HepG2 cells that occurs at 72 hour time point of post-infection period. Models of persistent chlamydial infection might be required for evaluating hepatic LDL-receptor turnover in the infected liver cells.

Secondly, we have clearly shown that mevastatin, an inhibitor of cholesterol biosynthesis, restores LDL-receptor mRNA and has a significant anti-chlamydial activity reducing chlamydial growth in infected hepatocytes. Genome of *C. trachomatis *does not contain genes responsible for lipid biosynthesis. Chlamydial species are known to acquire cholesterol, fatty acids and triglycerides from the host cells [26]. Therefore, it was reasonable to believe that targeting the cholesterol biosynthetic pathway in the host cells might affect chlamydial infection rate. This prediction was confirmed by RT PCR analysis. It is well acknowledged, that *C. trachomatis *16S rRNA gene expression is an informative criterion of chlamydial developmental cycle expressed in both early and late stages of *C. trachomatis *infection [27]. Detection of 16S rRNA transcript as a marker of viable and metabolically active *Chlamydia *allows to evaluate the effectiveness of different antibacterial agents [28]. Maximum inhibition of 16S rRNA as well as drastic reduction in the number of infected immunofluorescence-positive cell has been seen at 40 μM mevastatin level. Less pronounced decline in 16S rRNA transcript level has been observed at 20 μM mevastatin concentration. Even though addition of 20 μM mevastatin did not result in complete inhibition of chlamydial growth in HepG2 cells, there was formation of smaller chlamydial inclusions. Those are often observed in antibiotic- and/or cytokine-treated cells when concentration of the agent is not enough to induce complete eradication of the pathogen [23]. "Aberrant" chlamydial cells are known to have some metabolic activity but fail to induce new rounds of chlamydial infection [23,28]. Therefore inhibition of chlamydial growth in mevastatin-treated HepG2 cells takes place in clearly dose-dependent manner. Step-wise decline in 16S rRNA level was accompanied by reduction in the number of infected cells (1 and 20 μM mevastatin), as well as the appearance of "aberrant" chlamydial forms (20 μM mevastatin) until complete eradication of chlamydial growth takes place (40 μM mevastatin). Euo mRNA level has been changing in a similar manner, except inconsistent increase seen at 20 μM concentration of mevastatin. However, it is known that euo mRNA can be highly induced when the developmental cycle of *C. trachomatis *in cultured cells is compromised by addition of cytokines and other substances affecting chlamydial growth [28]. It has been proposed, that increased expression of euo may inhibit transcription of the genes specific for "late phase" of chlamydial developmental cycle [28,29]. Thus, enhanced transcription rate of euo may represent self-sufficient mechanism predetermining anti-chlamydial activity of mevastatin.

It is also important to conclude, that according to our results mevastatin has no effect on initial interaction of chlamydial particles with host cell, allowing the entry of the pathogen into hepatocytes. Therefore we assume that later stages of chlamydial developmental cycle are affected by mevastatin treatment. The effect of different metabolites and inhibitors of mevalonate pathway needs to be tested in hepatocytes infected with *C. trachomatis *in presence of mevastatin. It is possible, that anti-chlamydial activity of mevastatin takes place due to reduced geranylgeranylation of host cell proteins as it happens in case of lovastatin-treated hepatocytes infected with hepatitis C virus [30].

## Conclusions

We have demonstrated that ongoing cholesterol synthesis is essential for chlamydial growth in hepatocytes. Although the precise mechanism of anti-chlamydial activity of mevastatin remains to be elucidated, targeting the cholesterol biosynthetic pathway may represent an effective strategy in management of chlamydial infection.

## Competing interests

The authors declare that they have no competing interests.

## Authors' contributions

YKB and NAZ contributed equally into design, acquisition of data, analysis and interpretation of the results. YPP and LNK performed immunostaining and RNA protocols. IMP contributed into primary concept, drafting the manuscript, and final approval for publishing the results. All authors read and approved the final manuscript.
